# Brokering or Sitting Between Two Chairs? A Group Perspective on Workplace Gossip

**DOI:** 10.3389/fpsyg.2022.815383

**Published:** 2022-07-08

**Authors:** José Luis Estévez, Károly Takács

**Affiliations:** ^1^Department of Management and Engineering, The Institute for Analytical Sociology, Linköping University, Norrköping, Sweden; ^2^Department for the Study of Religions, Centre for the Digital Research of Religion, Masaryk University, Brno, Czechia; ^3^Computational Social Science – Research Center for Educational and Network Studies (CSS-RECENS), Centre for Social Sciences, Budapest, Hungary

**Keywords:** workplace gossip, organizational networks, informal groups, brokerage, multilevel analysis

## Abstract

Brokerage is a central concept in the organization literature. It has been argued that individuals in broker positions—i.e., connecting otherwise disconnected parts within a firm’s social network—can control the flow of information. It would imply their increased relevance in workplace gossip. This allegation, however, has not been addressed empirically yet. To fill this gap, we apply social network analysis techniques to relational data from six organizations in Hungary. First, we identify informal groups and individuals in broker positions. Then, we use this information to predict the likelihood with which positive or negative gossip is reported. We find more gossip when the sender and receiver are part of the same group and more positive gossip about in-group rather than out-group targets. Individuals in broker positions are more likely the senders and targets of negative gossip. Finally, even if both the brokers and the boss(es) are the targets of their colleagues’ negative gossip, the combination of the two categories (bosses in broker positions) does not predict more negative gossip anymore. Results are discussed in relation to the theoretical accounts on brokerage that emphasize its power for information control but fail to recognize the pitfalls of being in such positions.

## Introduction

During the last few years, organization researchers have shown an increasing interest in *workplace gossip* (Kniffin and Wilson, 2005; Michelson et al., 2010; Mills, 2010; Kong, 2018; Kuo et al., 2018; Wu et al., 2018; Beersma et al., 2019; Dores Cruz et al., 2019b; Fan and Grey, 2020; Lee and Barnes, 2021; Spoelma and Hetrick, 2021; Tan et al., 2021; Zong et al., 2021). One reason for this attention lies in the acknowledgment that gossip is a behavior that can have both “bright” and “dark” effects on a firm’s dynamics (Brady et al., 2017; Dores Cruz et al., 2019b). On the one hand, gossip can wreck the image of some individuals, affecting their performance, commitment, or self-esteem (Wu et al., 2018; Xie et al., 2019; Martinescu et al., 2021). On the other hand, gossip can sustain mechanisms fostering cooperation, mutual control, and self-organization (Ellickson, 1991; Dunbar, 1998; Hardy and Van Vugt, 2006; Nowak, 2006; Barclay and Willer, 2007; Piazza and Bering, 2008; Beersma and Kleef, 2011; Feinberg et al., 2014; Wu et al., 2016; Boehm, 2019; Giardini and Wittek, 2019a; Számadó et al., 2021; Giardini et al., 2022).

We define “workplace gossip” as any form of communication in which one member of the organization (the sender) provides another (the receiver) with evaluative information about an absent third (the target). Note that this definition characterizes gossip as a three-person phenomenon. Also, it can contain both positive and negative content. Although multiple conceptualizations of gossip exist (for a review, see Dores Cruz et al., 2021a), our definition is in line with typical usage in the literature: a communication about someone in their absence (hence, unaware of the communicated content). The three parties would allow us to distinguish between *emission* (or sending), *reception*, and *being the target* of somebody else’s gossip. The inclusion of *positive and negative content* enables comparing two related yet dissimilar forms of third-party communication. Examples of positive gossip may be praising, defending, or vouching for an absent colleague, whereas negative gossip could be blaming, criticizing, or complaining about them.

Since gossip can be consequential for the functioning of an organization, multiple studies try to identify which factors favor (or hinder) this behavior. Two research strands stand out in the literature. The first one looks primarily into the multiple functions gossip serves for those engaging in it. To this day, the list of motivations includes information gathering and validation, social influence, interpersonal aggression, emotion venting, social enjoyment, group protection, social bonding, clarifying norms, social comparison, and status enhancement (Dunbar, 2004; Wert and Salovey, 2004; McAndrew et al., 2007; Beersma and Kleef, 2012; Hartung et al., 2019; Shank et al., 2019; Dores Cruz et al., 2019a). The second strand underscores structural aspects instead as the drivers of gossip, for instance, interdependencies (e.g., competition and collusion) and informal social networks (Wittek and Wielers, 1998; Grosser et al., 2010; Ellwardt et al., 2012a; Giardini and Wittek, 2019b; Yucel et al., 2021). These two strands should not be seen as opposed, however. For example, a motivation to harm someone’s reputation may be the presence of an underlying negative relationship tie (e.g., envy, dislike, distrust). So-called coalition triads (Wittek and Wielers, 1998) can result from social bonding where two individuals grow closer by expressing shared animosity for a specific target (Bosson et al., 2006; Peters et al., 2017).

This article aims to contribute to the literature on workplace gossip by focusing on two structural aspects that have received scant attention: *informal groups* and *broker positions*. By informal groups, we refer to relatively cohesive communities that stand apart from each other (Stadtfeld et al., 2020) and exist more or less independently from the formal organizational structure (i.e., work teams). Brokerage is a central concept in the organization literature (Gould and Fernandez, 1989; Burt, 1992, 2007). It has been argued that individuals in broker positions—namely connecting otherwise disconnected parts of an organization—may have control over the information flow in a company (Stovel and Shaw, 2012; Kwon et al., 2020), which would imply their increased relevance in organizational gossip. This claim, however, has not been addressed empirically yet (Foster and Rosnow, 2006).

In the next section, we develop expectations regarding how membership in the same group and having a broker status can affect the dynamics of workplace gossip (i.e., who gossips with whom about whom). Hypotheses are tested using data collected in six different working units (*N* = 128), all located in Budapest (Hungary). Network data were collected and then transformed using composite networks (Vörös and Snijders, 2017) and graph partitioning (Blondel et al., 2008) to identify informal groups within each unit. Individuals with a broker status were singled out based on their betweenness centrality (Freeman, 1977, 1978). Multilevel models (Snijders and Bosker, 2011) connected same-group membership and having a broker status with the gossip reported: who gossiped with whom, about whom, and how (positively vs. negatively).

## Theoretical Framework

### Structural Antecedents of Gossip

According to previous studies, workplace gossip is a behavior whose occurrence and valence (positive vs. negative) are shaped by structural dimensions, in particular by underlying relationship ties (e.g., friendship, liking, enmity, trust; Turner et al., 2003; Grosser et al., 2010; Dores Cruz et al., 2021b). Since gossip requires at least three people in different roles (sender, receiver, and target), “structural antecedents” comprise the three ties within the gossip triad (Wittek and Wielers, 1998; Giardini and Wittek, 2019b): the *sender–receiver*, the *sender–target*, and the *receiver–target* relations. We address each of these ties in detail below:

First, a good relationship (e.g., personal closeness, affection, alliance, or trust) is usually invoked as a critical condition facilitating the sharing of gossip (Bergmann, 1993; Gambetta, 1994; Burt, 2001). Gossip always entails talking behind someone else’s back, which is a *socially condemned behavior* in almost all cultures (Foster, 2004; Giardini and Wittek, 2019b). One way of maneuvering around this social norm is sharing gossip selectively—the more sensitive the content, the more exclusively (e.g., with friends and acquaintances vs. with close friends only). Grosser et al. (2010) obtained support for this idea using social network data collected in an American firm. They observed that an expressive tie (e.g., friendship) between the sender and the receiver predicts negative gossip, whereas instrumental ties suffice for the exchange of positive gossip.

Second, while a positive tie between the sender and receiver facilitates gossip sharing, a negative relation between the sender and target can be the driver behind negative gossip. Spreading negative gossip about someone can be a form of *relational aggression* (Ingram, 2014; McAndrew, 2014; Davis et al., 2019). Not surprisingly, individuals would preferably spread negative gossip about enemies and rivals (McAndrew et al., 2007; Davis et al., 2019; Hess and Hagen, 2019; Wyckoff et al., 2019). Positive gossip, in contrast, can be *status enhancing* for the target. For this reason, most people refrain from passing along positive information about enemies and rivals and do it instead about those with whom they have a good relationship (e.g., friends or allies; McAndrew et al., 2007).

Lastly, the tie between the gossip receiver and the target is probably the most subtle of the three. The moment a person decides to pass along information about somebody else; it is to be expected that they will first assess the connection between their potential recipient(s) and the target (Burt and Knez, 1995; Burt, 2001). If these two have a good relationship, negative information is likely withheld. In organizations, *selective disclosures* are observed in the way employees complain about colleagues (Behfar et al., 2019). Hostility between the receiver and target can give cause for negative gossip, even when the sender might not feel animosity toward the latter. This effect is detected among adolescents in classrooms (Estévez et al., 2022). One explanation for this pattern is that (mutual) negative ties offer a perfect chance to share the gossip that *strengthens social bonds* (Dunbar, 1998, 2004; Bosson et al., 2006; Peters et al., 2017).

In sum, the underlying relationship ties among the three gossip parties have proven to play a crucial part in gossip dynamics (i.e., who gossips with whom, about whom, and how). Hereafter, we will focus on two additional structural aspects: *informal groups* (the cohesive communities that stand apart from each other and exist more or less independently from the formal organizational structure) and *brokers* (individuals whose social connections cut across groups bringing together distant parts of an organization). “Gossip and Informal Groups” explains why informal groups are relevant for workplace gossip, whereas “Gossip and Brokerage” addresses how individuals in brokerage positions may affect the dynamics of workplace gossip.

### Gossip and Informal Groups

A close association between gossip and informal groups is not new in the gossip literature (Gluckman, 1963; Merry, 1984; Elias and Scotson, 1994; Michelson et al., 2010). Gluckmann already expressed this core intuition in what is considered one of the first scientific studies on gossip:

‘[Gossip] is a privilege which is only extended to a person when he or she is accepted as a member of a group […] it is a hallmark of membership.’ (Gluckman, 1963, p. 277).

According to Gluckmann, what makes a person a genuine group member is the decision of other group fellows to extend the gossip with them. Curiously, Gluckmann’s argument lays bare an aspect often downplayed in the literature: whereas one can always distinguish between a sender, a receiver, and a target analytically; in practice, gossip is often *expressed in small groups* (Hannerz, 1967; Elias and Scotson, 1994; Kurland and Pelled, 2000; Kniffin and Wilson, 2010).

In the workplace, informal groups are characterized by *intense interaction* among their members (e.g., cooperating on tasks, sharing lunch or dinner after work), which favors engagement in gossip. On top of more chances for gossip to happen, informal groups also create the *expectation* (if not the *obligation*) to do it. For instance, it raises suspicion if individuals are part of a group but avoid sharing gossip with their group fellows. These may feel that critical information is withheld for some shadowy reasons (Nijstad and De Dreu, 2012; Levine and Smith, 2013) or that this member’s loyalty does not lie in the group but elsewhere. As a general pattern thus, one can expect that most gossip in an organization would be shared within informal groups: with other members of one’s informal group rather than with non-members.

People are expected to share gossip *with* other members of their informal group rather than with non-members. This holds for positive gossip (H1a) and negative gossip (H1b).

Other members of the informal group can be not only the natural recipients of gossip but also their targets. Previous research observed that both positive workplace gossip and negative workplace gossip focus on members of the sender’s work team (Kniffin and Wilson, 2005; Ellwardt et al., 2012a). One reason why positive gossip concentrates on group members is that this gossip helps develop and sustain in-group solidarity norms (Dunbar, 2004):

‘By gossiping positively about other members of our group who are not present, group members stay informed about each other, and demonstrate support and solidarity towards the gossip object and the group’ (Ellwardt et al., 2012a, p. 195).

In addition, sending positive gossip about co-members can also improve the sender’s reputation by signaling a commitment to in-group norms (Ellwardt et al., 2012a). All in all, since positive gossip about group members can yield potential benefits both for the sender (*status-enhancement*) and the group (*solidarity norms*), the expectation is that it will occur among in-group members and refer to another member of the same group. The above implies that people would also be less inclined to send/receive positive gossip about individuals who are not members of their informal group.

According to previous studies (Kniffin and Wilson, 2005; Ellwardt et al., 2012a), negative gossip concentrates on same-team members because individuals in teams are *interdependent* (e.g., bonuses and other benefits can depend on collective performance). Consequently, violations committed by other team members are more important and judged more sternly. This argument, however, downplays the high *costs of negative gossip* in informal groups, where individuals are affective rather than functionally interdependent (Giardini and Wittek, 2019b).

One of the main reasons people share negative gossip at work is not because they want to manipulate someone else’s reputation but because of its strong bonding effect with other individuals (Dunbar, 2004; Bosson et al., 2006; Peters et al., 2017). In other words, sharing negative gossip can be just a form of social glue (Turner et al., 2003). For this purpose, however, the sender must select a target without hurting the receiver. It is well established that exposure to gossip that confronts our positive opinion of a specific person tends to elicit a *negative response* (Hallett et al., 2009; Caivano et al., 2020): the sender might lose face in the eyes of the receiver, be perceived as unable to solve their problems, be vindictive, or just evil. Gossip can even *trigger a conflict* between the sender and the receiver (Giardini and Wittek, 2019b).

As a result of the above, we expect that most people would temper their inclination to share negative impressions of someone too close to their receivers (e.g., another group member). Instead, one way of exploiting the bonding effects of negative gossip is talking about relatively distant others. Previous studies have noticed that stereotypical persons and stigmatized minorities are exceptionally functional for this (Crothers et al., 2009; Carrim, 2016). Also, preferentially shared negative gossip have been used to explain why individuals’ reputations can be sticky, especially in dense networks (Burt, 2001, 2008).

To sum up, we argue that because of its potential benefits for the sender (status-enhancement) and the group (solidarity norms), most positive gossip focuses on targets who are members of the same group as the sender and receiver. In contrast, negative gossip will focus on out-group targets instead to prevent hazards like face loss or conflict escalation.

People are expected to *send* positive gossip *about* other members of their informal group rather than about non-members (H2a), whereas they are expected to send negative gossip about out-group members rather than in-group members (H2b).

People are expected to *receive* positive gossip *about* other members of their informal group rather than about non-members (H3a), whereas they are expected to receive negative gossip about out-members rather than in-group members (H3b).

Notice that, unlike in previous work, we distinguish here between emission and reception as two closely related yet different dimensions of gossip.

### Gossip and Brokerage

Thus far, we talked about informal groups as if their members were perfectly outlined (either someone is a member or is not). Often, however, we observe that some individuals, because of their patterns of interactions with other colleagues, could be part of several groups (Krackhardt, 1999; Vedres and Stark, 2010; Tasselli and Kilduff, 2017). This fact gives cause for another differentiation: individuals whose connections are primarily within the same group vs. individuals whose ties cut across different groups. Organizational and social scholars sometimes refer to these individuals who bring separate parts of a network together as (network) “brokers” (Gould and Fernandez, 1989; Burt, 1992). For conciseness, we will also use this term here. Curiously, although an extensive literature has connected having a broker position with multiple benefits derived from information advantages (Burt, 1992, 2004, 2010; Stovel and Shaw, 2012; Kwon et al., 2020), the link between workplace gossip and having a broker status remains almost entirely unaddressed.

Foster and Rosnow (2006) argued that individuals in broker positions might feel more compelled to employ negative gossip insofar as, compared to those in more embedded positions, the opportunity structure they face allows for *exploiting personal benefits*. Specifically, it gives the broker a competitive advantage that their potential receivers often belong to separate groups. As long as the individuals in those different groups seldom interact, brokers can manipulate the information they share to their benefit with low risk of others will cross-check its veracity.

Of course, having the opportunity to exploit their position and actually doing it are two different things (Halevy et al., 2019). Notwithstanding this, previous studies have observed how, in organizations at least, individuals in bridging positions also tend to present certain traits, like *self-monitoring personalities* (Sasovova et al., 2010; Landis, 2016). Considering that brokers may extract more considerable gains from negative gossip and that they tend to present personalities that make them more in need of others’ attention and approval, a foreseeable pattern is that, in the workplace, brokers send negative gossip on a more regular basis:

Brokers are expected to send more negative gossip than individuals whose ties are primarily intra-group (H4).

Whereas the argument of Foster and Rosnow (2006) pertains to the emission of gossip, they did not address how brokers may receive or be targets of others’ gossip. Indeed, it is well known that having a broker status might provide an advantage for information access and control (Burt, 2004; Stovel and Shaw, 2012; Kwon et al., 2020). Nonetheless, bridging positions between informal groups can also come with high costs. In a seminal work, Simmel (1950) already underlined how go-betweens could be left with a sense of anomie since they are *not full-fledged members of any group*. When informal groups are the basis of a strong ‘us-them’ mentality (e.g., due to assortative mixing, past conflicts, or intense group-level competition), group spanning can entail even larger disadvantages (Krackhardt, 1999; Tasselli and Kilduff, 2017). For example, members of the two (or more) groups that they bridge can perceive the broker with *suspicion* and *distrust*. In such cases, one may expect that brokers would be systematically avoided as confidants and, consequently, gossip partners. In addition to refraining from sharing gossip with them, suspicion and distrust can also cause brokers’ actions to be more thoroughly examined and frowned upon. The natural outcome thus is that many colleagues can share negative gossip about those in broker positions. In summary:

Brokers are expected to receive less gossip than individuals whose ties are primarily intra-group. This holds for both positive (H5a) and negative gossip (H5b).

Compared to those whose relationships are mainly within a group, brokers are expected to be more often the targets of others’ negative gossip (H6).

## Data, Measures, and Methods

### Research Setting

We collected data from six working units located in Budapest (Hungary) to test our hypotheses. The six units are independent. Three units are small-size companies, whereas the other three are subunits (departments) of a larger firm:

- Unit A (*N* = 24) is a subunit of a firm operating in the public sector. The personnel comprise a chief manager plus five other managers, each in charge of a team of three to seven. Employees are primarily social workers and administrative professionals, mostly young or middle-aged women.
- Unit B (*N* = 19) is a small web development company composed of six project managers (three women), one administrative professional, and twelve employees.
- Unit C (*N* = 29) is a subunit of a software development firm. All but one member of this unit are men. Of these, four hold management positions.
- Unit D (*N* = 18) is a firm working on the development of access control systems. The personnel in this unit is mostly engineers and IT specialists. Six (one woman) hold a management position.
- Unit E (*N* = 16) is a subunit of a different software development company. This unit comprises six men (all managers) and ten women (two in management positions).
- Lastly, Unit F (*N* = 22) is a firm operating in the financial sector. The personnel of this unit is mostly composed of middle-aged men, of whom four have managerial positions.

All units together, our sample comprises 87 men (68.0%) and 41 women (32.0%). The gender composition is very disparate across units, ranging from 75% women in Unit A to 3.4% in Unit C.^1^

Data were collected using self-administered computer-based questionnaires.^2^ First, we initiated personal contact with the CEOs. After obtaining their agreement, both managers and employees in the units were asked to participate in the survey. All information was collected between 2016 and 2018. Only 4 out of the 128 respondents did not complete the questionnaire (3.1% missing data).

### Measures

#### Response Variable: Gossip

Workplace gossip was collected by asking respondents three nested questions. First, each respondent was requested to indicate who of all their colleagues (*sender*) provided them with personal information about another colleague while the latter was absent from the conversation. After this, for each sender declared, respondents were asked to report who the object of the gossip was (*target*). Finally, for each pair sender–target, respondents had to characterize the tone of the information received as either positive, negative, or neutral (*valence*).

This tool is inspired by previous studies using the exact three-step procedure (Ellwardt et al., 2012b,c). Notice that the word ‘gossip’ was not used in the questionnaire. Since the term carries negative connotations, we avoided its usage to prevent non-response. This data collection procedure provides us with a number of “rated gossip triplets” ( $g_{\mathrm{srtv}}$ ) per unit, where 𝑠 stands for the gossip sender, 𝑟 for the respondent in the role of receiver, 𝑡 for the target, and 𝑣 for the valence of the contents. For analysis, these gossip triads are transformed into two dummy variables—*positive gossip* and *negative gossip*—as we explain in “Method.”

#### Explanatory Variables: Same-Group Membership and Brokerage

Same-group membership and whether an individual has a broker status were computed using standard social network analysis tools.

The *Louvain algorithm* for graph partitioning (Blondel et al., 2008) was used to divide each of the six working units into smaller non-overlapping communities (here, informal groups). Graph partitioning comprises a family of algorithms, all of which use a set of relationship ties among a finite number of actors to uncover latent community structures, like cliques or groups of friends (Newman and Girvan, 2004; Blondel et al., 2008; Lancichinetti and Fortunato, 2009; Bruggeman et al., 2012). The solution given by the Louvain algorithm was used to create a dyadic-level variable ( $X_{ij}$ ) capturing where two individuals ( i , j ) are members or not of the same group: $x_{ij}=1$ indicates that i and j are group fellows, otherwise $x_{ij}=0$ .

To classify individuals as brokers or not, we used *betweenness centrality*—a measure of the extent to which an actor serves as a potential go-between for other pairs of actors (Freeman, 1977, 1978). As with graph partitioning, this procedure requires a set of relationship ties defined over a finite number of individuals. However, the outcome here is not a subset of actors but a value for each individual summarizing their “brokerage potential.” These values were dichotomized as either “1” (broker) or “0” (non-broker) in a second step by running a hierarchical clustering (Borgatti et al., 2013) on the matrix of absolute distances for every pair of individuals. Notice that *broker* is an individual-level variable ( $X_{i}$ ), unlike *same group* which is a dyadic-level variable ( $X_{ij}$ ).

**Figure 1:**
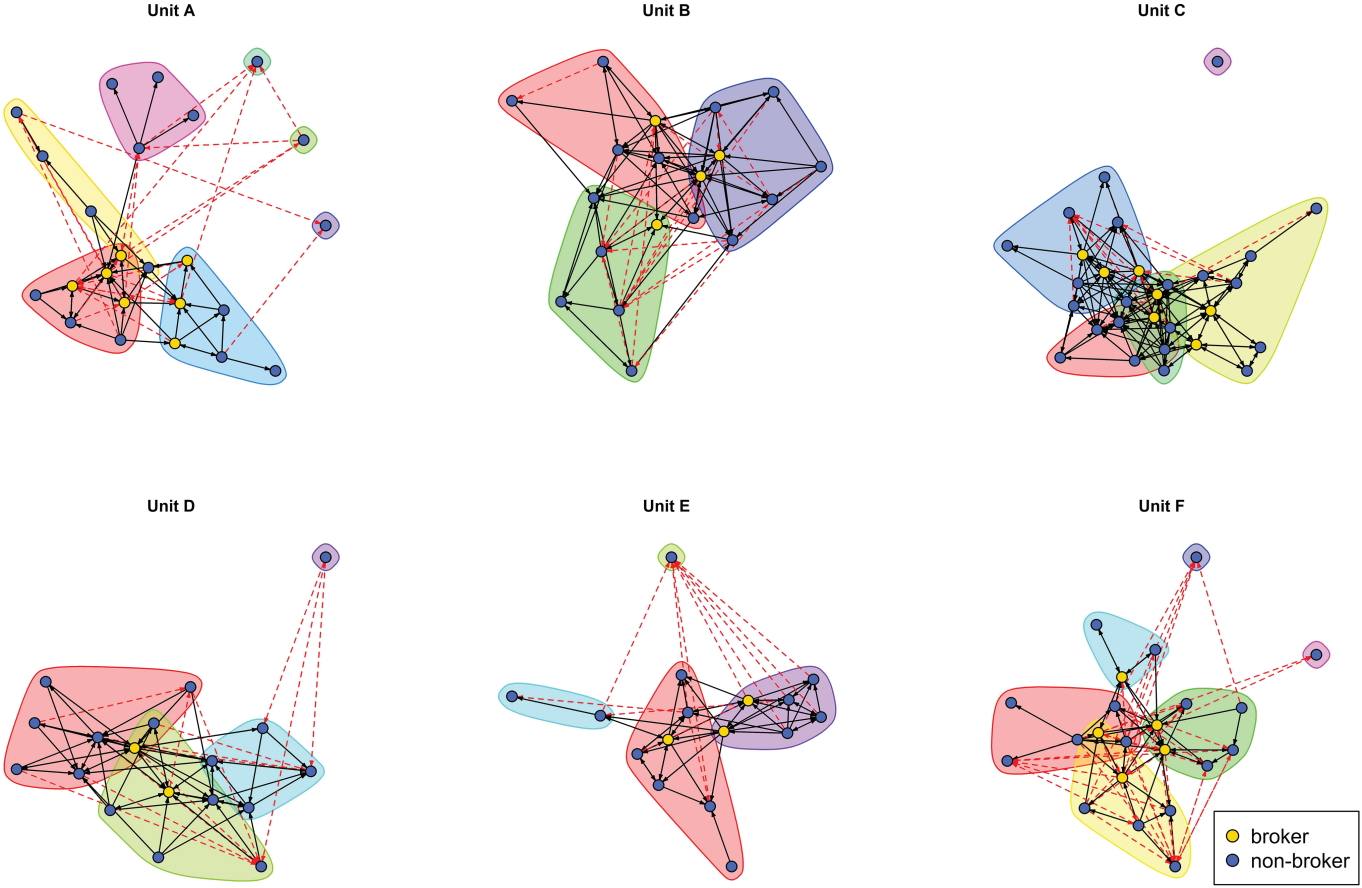
Visual representation of the informal groups and brokers in every unit. Solid black arrows represent positive ties. Dashed red arrows represent negative ties. Colored areas in the background capture different informal groups. Individuals with a broker status are colored in yellow, non-brokers in blue.

While the input of these two procedures has not been explained yet (the network of positive ties; see “Control Variables”), Figure 1 visually displays the output in every unit. Isolates aside (individuals with no positive ties with anybody else), each unit contains three-to-four informal groups. The number of brokers is heterogenous across working units, ranging from only two in Unit D to seven in Units A and C. In total, 28 of our 128 individuals were classified as ‘brokers’ (21.9% of the sample).

#### Control Variables

##### Gender

Controlling for gender responds to the commonly held belief that women are more gossipy than men (Michelson and Mouly, 2000). Women are identified with “1” and men with “0” in our data.

##### Hierarchical Position

Individuals holding positions of responsibility in a company may have a more significant degree of involvement in the office grapevine. One can expect that many employees actively seek information about those they depend upon and whose decisions can affect them most (Ellwardt et al., 2012c). Further, managers and team leaders can activate “tall poppy syndrome” (Feather, 1998), making others find pleasure in pulling them down (Graffin et al., 2013). As a result of all this, gossip about bosses is likely to be more widespread than gossip about mere employees. We created a dummy variable where “1” stands for being a boss to control this potential bias. Otherwise, the value is “0.” As a boss, we include any person whose title in the firm contains the words “manager,” “director,” “leader,” or “chief.”

##### Positive and Negative Relationship Ties

As mentioned before (“Structural Antecedents of Gossip”), underlying relationship ties like who has a good (or bad) relationship with whom (Wittek and Wielers, 1998; Ellwardt et al., 2012a; Yucel et al., 2021; Estévez et al., 2022) is one of the main predictors of gossip. These relationships, however, are not directly observable and need to be inferred somehow. Given the small size of all units, we collected sociometric data on 25 dimensions (e.g., friendship, trust, appreciation), including impressions and assessments (e.g., whether the other person is popular, does their job well, or deserves a salary raise or cut). Then, we used all these items to construct two binary networks (one of “positive ties” and the other of “negative ties”) in a data-driven fashion.

**Figure 2:**
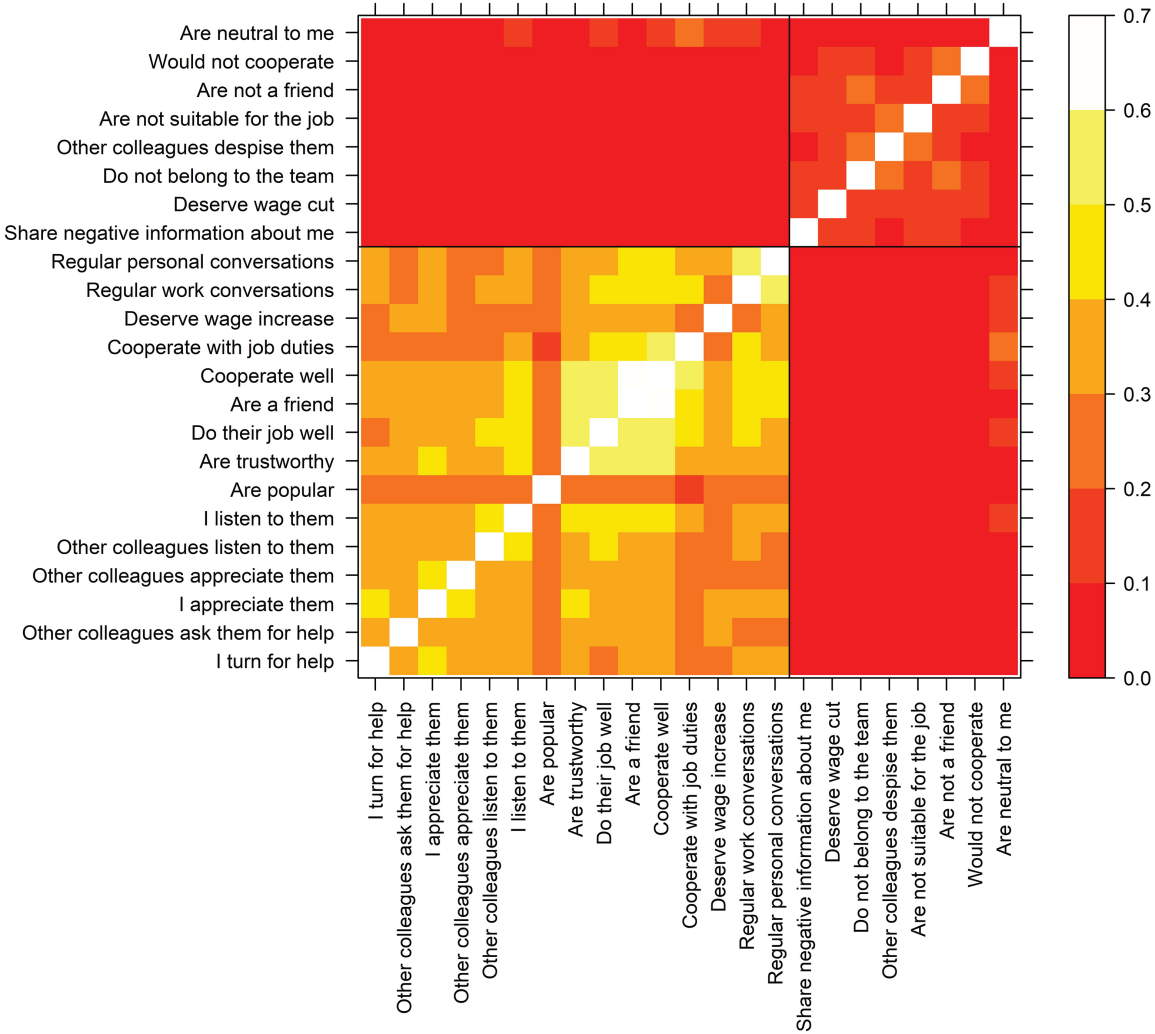
Overlap between network items (mean Jaccard indices for all six units).

The procedure followed the guidelines proposed by Vörös and Snijders (2017). First, we calculated the matrix overlap (Jaccard index) between each pair of items per unit and checked the consistency of these values across units. Iteratively, items with a Kendall W below 0.5 were excluded, meaning that the resemblance of these items across units was poor.^3^ With the remaining items, we search for latent dimensions based on the similarity of the items in all six units (mean Jaccard indices). As Figure 2 shows, two latent dimensions emerged. On the bottom left corner of the figure, we can see 15 items with a resemblance ranging from 19.3% up to 67.6%. All these items capture some positive relationship, impression, or assessment. On the top right corner, we see the remaining eight items. Here, overlaps are smaller (0.3–26.3%). Still, all these items share that they manifest negativity (or indifference at best). We chose a two-dimension solution because the interpretation of a two-cluster solution was straightforward (positive vs. negative).^4^

Lastly, we turned the two clusters of items detected into binary network variables ( $X_{ij}$ ): “positive ties” and “negative ties.” The transformation was done by adding up all matrices in the same cluster into a “weighted” matrix ( $W_{ij}$ ) first. Then, we set a minimum number of nominations as a threshold. If $w_{ij}\geq \mathrm{threshold}$ , then $x_{ij}=1$ . Otherwise $x_{ij}=0$ . Concretely, we used 10 out of 15 for the positive cluster and 3 out of 8 for the negative one. These threshold values were chosen to yield relatively low out-degrees.^5^ Also, they do not create overlaps between the resulting positive and negative ties (i.e., no person in our sample nominates someone else positively and negatively).^6^

The resulting positive and negative ties are shown in Figure 1. Besides considering them as a control variable, the positive ties were used to identify informal groups and brokers in the work units (as explained in “Explanatory Variables: Same-Group Membership and Brokerage”).

### Method

Multilevel models (Snijders and Bosker, 2011) connected our response and explanatory variables. First, we retrieved all possible permutations of three individuals per working unit to create the sample space: $N\left(N-1\right)\left(N-2\right)$ . Then, we created two dichotomous triadic-level variables ( $X_{ijk}$ ) where we allocated the rated gossip triplets ( $g_{\mathrm{srtv}}$ ). Specifically, in the variable *positive gossip*, $x_{ijk}=1$ indicates that person i sent positive gossip to j about target k . If not, $x_{ijk}=0$ . The same goes for the second variable, *negative gossip*, where $x_{ijk}=1$ indicates that person i sent negative gossip to j about k , otherwise $x_{ijk}=0$ . The treatment of positive and negative gossip as two different variables responds to the fact that we hypothesized opposite effects in the patterns of targeting depending on the gossip valance (see H2a and H2b, for instance). If combined together, some factors could cancel each other out. Notice that we did not analyze gossip characterized as neutral. The reason is that, unlike other scholars (Robbins and Karan, 2020; Dores Cruz et al., 2021a,b), we excluded exchanges with non-evaluative contents from our definition of gossip.

Because our models measure the contribution of individual-level and dyadic-level variables to the occurrence of specific triadic configurations ( $x_{ijk}=1$ ), we always input one predictor in several different ways. For individual-level variables ( $X_{i}$ ), we include three variants of the same variable capturing the contribution of the factor in question to the three gossip roles. For example, we have a predictor for the gender of the gossip sender [*woman* (*sender*)], receiver [*woman* (*receiver*)], and target [*woman* (*target*)]. For dyadic-level variables ( $X_{ij}$ ), we use dyadic combinations, for instance, whether the sender and receiver share a positive tie [*positive tie* (*sender–receiver*)], the sender and target [*positive tie* (*sender–target*)], or the receiver and target [*positive tie* (*receiver–target*)].To account for the cross-nested structure of our data, we built our models using examples handing triadic network data as inspiration (Bond et al., 1997; Card et al., 2010; Van Duijn, 2011; Swartz et al., 2015). Concretely, we let $\theta_{\mathrm{ijku}}$ be our response variable, namely the probability of observing a specific positive (or negative) gossip triad in unit u , or $\mathrm{Pr}\left(\mathrm{gossi}p_{\mathrm{ijku}}=1|i\neq j,i\neq k,j\neq k\right)$ , assuming independent binary responses: $\mathrm{gossi}p_{\mathrm{ijku}}\sim \mathrm{Bernoulli}\left(\theta_{\mathrm{ijku}}\right)$ . Then, we modeled $\theta_{\mathrm{ijku}}$ as:


$$\mathrm{logit}\left(\theta_{\mathrm{ijku}}\right)=\mu +A_{u}+B_{iu}+C_{ju}+D_{ku}+\varepsilon_{\mathrm{ijku}}$$

where μ is the intercept or grand mean of the model. $A_{u}$ refers to the random variation in the intercept across working units. $B_{iu}$ , $C_{ju}\text{,}$ and $D_{ku}$ represent the random variation for the intercept across individuals in the roles of the sender, receiver, and targets, respectively. Finally, $\varepsilon_{\mathrm{ijku}}$ is the error term. Note that results are calculated for the six units altogether in order to guarantee statistical power for all the estimated parameters (some could not be estimated for each unit independently).

Models were fitted in four steps. First, we ran a null model that includes only random (non-fixed) factors to observe different sources of variability (namely, across units, senders, receivers, or targets). Second, we added the control variables: gender (*woman*), formal hierarchy (*boss*), *positive ties*, and *negative ties*. Third, we included all predictors related to membership in the *same group*. Lastly, we extended the previous model specification with the effects for *brokers*. Notice that we also included two extra dimensions. *Isolates* were included because they can distort the comparison between brokers and non-brokers (individuals whose connections are primarily within the same group). *Same group* (*sender–receiver–target*) is an interaction term seizing the effect when all three gossip parties are in the same group rather than in combinations of two (*sender–receiver*, *sender–target*, *receiver–target*).

Analyses were performed in the statistical system R (R Core Team, 2021), using the package *lme4* version 1.1–27-1 (Bates et al., 2015). Coefficients were standardized with the package *effectsize* version 0.6.0.1 (Ben-Shachar et al., 2020), and marginal and conditional *R*^2^ values were computed using the package *insight* version 0.17.1 (Lüdecke et al., 2019). In the article, we only report standardized estimates for the fixed effects. For further results, the reader can see Supplementary Tables S5 and S6.

## Results

### Descriptive Results

Table 1 displays the summary statistics of our gossip data. Remember that gossip here is in the form of valued triplets ( $g_{\mathrm{srtv}}$ ). All six units together, 557 positive and 446 negative unique gossip triads were reported. These figures represent roughly 1.0 and 0.8% of all possible triplets (in the hypothetical scenario where every respondent had received gossip from everyone in the office about everyone else). More positive than negative triads are observed in units B, C, D, and E. In contrast, there are more negative than positive gossip triads in Units A and F. This is probably related to the presence of more negative relationship ties (and fewer positive ties) in these two units.^7^

**Table 1:** Summary statistics of gossip.

	Unit A	Unit B	Unit C	Unit D	Unit E	Unit F	Total
**Gossip triads**							
Positive gossip triads	79	94	157	66	123	38	557
	(0.65%)	(1.62%)	(0.80%)	(1.35%)	(3.66%)	(0.43%)	(1.02%)
Negative gossip triads	144	37	38	32	85	110	446
	(1.19%)	(0.64%)	(0.19%)	(0.65%)	(2.53%)	(1.25%)	(0.82%)
Potential triads (NA excluded)	12,144	5,814	19,656	4,896	3,360	8,820	54,690
**Individuals**							
Unit members	24	19	29	18	16	22	128
Gossip reporters	15	9	17	11	10	12	74
**Positive gossip**							
Senders	17	12	18	13	15	13	88
Receivers	14	9	14	8	8	7	60
Targets	21	19	29	16	15	16	116
**Negative gossip**							
Senders	17	10	14	9	15	15	80
Receivers	12	8	7	9	8	8	52
Targets	22	8	18	10	14	22	94

Taken together, 74 of the 128 respondents (57.8%) reported at least one gossip triplet. Eighty-eight individuals (68.8%) were reported as senders in at least one positive gossip triad and 80 (62.5%) as senders of negative gossip. One hundred sixteen (90.6%) were reported as the target in at least one positive gossip triad and 94 (73.4%) as targets of negative gossip. If we disregard the valence of the gossip, 112 individuals (87.5%) were reported as either gossip senders or receivers, and 127 (99.2%) as gossip targets. Thus, all but a single subject was involved in the gossip triads collected in one of the three gossip roles (sender, receiver, target).

When we look at the interplay between gossip and same-group membership, in 45.6% of all positive gossip triads (254/557) and 32.0% of the negative gossip triads (178/446) are the sender and receiver members of the same group. In 230 of all positive gossip triads (41.3%) and 77 of all negative gossip triads (13.8%) are the sender and target in the same group. Similarly, in 195 of all positive gossip triads (35.0%) and 96 of all negative gossip triads (17.2%) are the receiver and the target group fellows. If we consider those cases where all three gossip parties are in the same group, they represent 20.5% of all the positive gossip triads (114/557) and 5.6% of all the negative gossip ones (5.6%). Overall, these numbers suggest that, compared to negative gossip, positive gossip is more likely shared among group members and focuses on other group members.^8^

**Figure 3:**
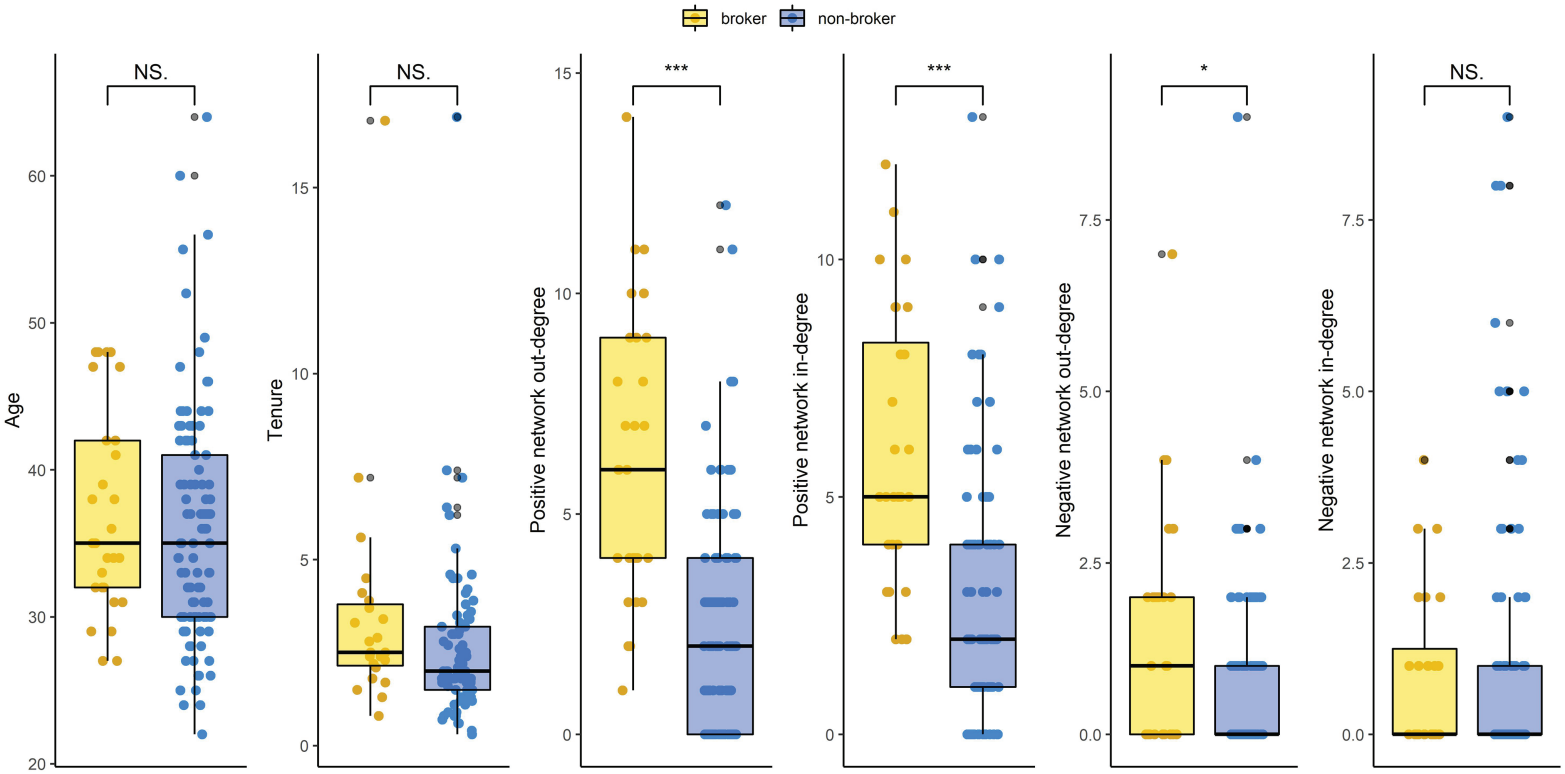
Comparison between brokers and non-brokers. NS. non-significant, *p < 0.05, **p < 0.01, and ***p < 0.001.

Before addressing the involvement of brokers in gossip, we inspect whether individuals qualified as brokers possess specific characteristics compared to the rest of the sample. No differences in gender are observed: 8 women and 20 men were categorized as brokers for 33 non-broker women and 67 non-broker men [ $\chi^{2}\left(1\right)=0.05,p=.830$ ]. Brokers, however, seem to be overrepresented in management positions: 47.1% of all bosses (16/34) were categorized as brokers compared to only 12.8% (12/94) of all employees [ $\chi^{2}\left(1\right)=15.23,p<.001$ ]. As Figure 3 shows, there are no differences between brokers and non-brokers in age, tenure, or the number of negative ties received. And yet, brokers receive more positive ties and send more positive and negative ones.

Finally, Table 2 describes the involvement of brokers in the three gossip roles. Overall, 41.8% of all positive triads (233/557) and 42.4% of all negative triads (189/446) have a broker as the sender; 48.3% of all positive triads and 36.1% of all negative triads have a broker as the receiver; and 23.0% of all positive gossip triads and 29.4% of all the negative gossip triads have a broker as the target. Since brokers only represent 21.9% of the total sample (28/128), at first sight at least, the involvement of brokers in all three gossip roles seems to be noteworthy.

**Table 2:** Involvement of brokers in the gossip triads.

	Positive gossip				Negative gossip			
	As sender	As receiver	As target	Gossip triads	As sender	As receiver	As target	Gossip triads
Unit A	52	30	29	79	95	68	71	144
Unit B	38	55	23	94	16	14	3	37
Unit C	78	52	42	157	12	11	11	38
Unit D	18	35	6	66	14	4	1	32
Unit E	33	77	23	123	31	55	19	85
Unit F	14	20	5	38	21	9	26	110
Total	233	269	128	557	189	161	131	446

### Hypothesis Testing

The main results of the study are displayed in Table 3. Models 1 and 2 show the same model specification. The only difference between the two models is that the first has *positive gossip* as the response variable, whereas the second has *negative gossip*.

**Table 3:** Multilevel logistic estimates of the association between same-group membership/brokerage and gossip.

	Model 1 (positive gossip)			Model 2 (negative gossip)		
	Est.	95% CI		Est.	95% CI	
Constant	−8.64	−9.46	−7.81	−9.26	−10.17	−8.34
**Individual-level control variables**
Woman (sender)	0.14	−0.17	0.45	0.04	−0.30	0.37
Woman (receiver)	0.00	−0.53	0.53	0.21	−0.32	0.75
Woman (target)	0.01	−0.18	0.19	−0.16	−0.41	0.09
Boss (sender)	0.43	0.16	0.70	0.44	0.15	0.73
Boss (receiver)	0.55	0.03	1.06	0.82	0.29	1.35
Boss (target)	−0.01	−0.18	0.16	0.24	0.03	0.46
Isolate (sender)	0.01	−0.30	0.33	0.03	−0.28	0.34
Isolate (receiver)	0.36	−0.16	0.89	−0.32	−0.92	0.29
Isolate (target)	−0.24	−0.47	0.00	0.16	−0.03	0.35
**Dyadic-level control variables**
Positive tie (sender–receiver)	0.45	0.34	0.57	0.32	0.18	0.45
Positive tie (sender–target)	0.26	0.16	0.37	−0.20	−0.36	−0.05
Positive tie (receiver–target)	0.24	0.14	0.35	−0.26	−0.41	−0.11
Negative tie (sender–receiver)	−0.03	−0.16	0.10	0.23	0.14	0.32
Negative tie (sender–target)	−0.15	−0.31	0.00	0.26	0.20	0.33
Negative tie (receiver–target)	−0.14	−0.27	−0.01	0.25	0.18	0.32
**Variables based on group membership**
Same group (sender–receiver)	0.28	0.16	0.39	0.27	0.15	0.39
Same group (sender–target)	0.25	0.15	0.35	−0.08	−0.23	0.06
Same group (receiver–target)	0.10	−0.05	0.26	0.13	−0.03	0.29
Same group (sender–receiver–target)	0.00	−0.11	0.11	0.05	−0.07	0.18
**Variables based on broker status**
Broker (sender)	0.56	−0.12	1.23	0.76	0.06	1.47
Broker (receiver)	0.87	−0.42	2.15	0.22	−1.09	1.54
Broker (target)	−0.11	−0.53	0.31	0.66	0.14	1.18
Observations	57,378			57,378		
Marginal *R*^2^	0.151			0.155		
Conditional *R*^2^	0.749			0.782		

Marginal *R*^2^ indicates the proportion of the model variance explained by the fixed effects only. Conditional *R*^2^ indicates the proportion of the model variance explained by the fixed and random parts.

Focusing on the hypothesized effects, Model 1 shows that it has a positive contribution to gossip when the sender and receiver are in the same group: *same group* [*sender–receiver;* $\hat{\beta }$ = 0.28, 95% CI (0.16, 0.39)]. This finding provides support for H1a, which posits that being group fellows favors the exchange of positive gossip. A similar pattern is observed in Model 2: $\hat{\beta }$ = 0.27, 95% CI (0.15, 0.39). This finding supports H1b, according to which negative gossip is more likely to be exchanged between group fellows than between individuals in different groups. Together, these two findings corroborate our general expectation that gossip is more likely expressed among group members.

To address the hypotheses regarding the importance of group membership for who the target of the gossip is (H2a, b and H3a, b), we look at the contributions of *same group* (*sender–target*), *same group* (*receiver–target*), and *same group* (*sender–receiver–target*). Save the effect of *same group* (*sender–target*) on positive gossip [ $\hat{\beta }$ = 0.25, 95% CI (0.15, 0.35)]; none of these predictors contribute to gossip. It entails that though respondents were more likely to send positive gossip about a target in the same group (which supports H2a), no differences between group members and non-members are observed for who the target of negative gossip is (going against H2b and H3b). Also, the fact that *same group* (*sender–target*), but neither *same group* (*receiver–target*) nor *same group* (*sender–receiver–target*) has an association with positive gossip suggests that group membership may affect gossip emission but not reception (contradicting H3a). Put differently, people could be more inclined to gossip positively about those in the same group but not necessarily with other group fellows.

Hypotheses 4–6 concern the association between having a broker position and workplace gossip. Model 2 evaluates H4 regarding the more active part of brokers in the spread of negative workplace gossip. Consistent with this hypothesis, we find that *broker* (*sender*) makes a positive contribution to negative gossip [ $\hat{\beta }$ = 0.76, 95% CI (0.06, 1.47)]. In terms of gossip reception, no differences are observed between brokers and non-brokers. As Table 3 shows, *broker* (*receiver*) has no association with either positive or negative gossip. These findings contradict H5a and H5b, respectively. Finally, H6 posited that brokers would likely be the objects of their colleagues’ negative gossip. The positive contribution of *broker* (*target*) in Model 2 [ $\hat{\beta }$ = 0.66, 95% CI (0.14, 1.18)] confirms this expectation. Together, the findings in this paragraph support that brokers may send more negative gossip than those who are more embedded in a group. At the same time, however, brokers are also more likely to be targets of others’ negative gossip.

Regarding the variables that worked as a control in the present study, no association was detected between *gender* and gossip (for either sending, receiving, or being its object). This lack of effect echoes some previous studies (Levin and Arluke, 1985; Leaperand and Holliday, 1995) denying that women are more gossipy than men.

In terms of *hierarchy*, our results reveal that bosses played a key part in the dynamics of gossip. In Model 1, both *boss* (*sender*) and *boss* (*receiver*) have a positive contribution, whereas all three factors [*boss* (*sender*)*, boss* (*receiver*), *boss* (*target*)] have a positive contribution in Model 2. The above entails that those in managerial positions might be sending and receiving gossip more often when compared to employees (Kuo et al., 2018). As expected, bosses also seem to be those whom others gossip negatively about (Ellwardt et al., 2012c). Since the overlap between being a boss and having a broker status was substantive in our sample (16 of our 28 brokers were also bosses), to discard the possibility that brokers were negative targets simply because of their formal position, we reran Model 2 including the interaction *broker* (*target*) *× boss* (*target*). Results confirmed that the contribution of these two variables is independent while, strikingly perhaps, their interaction is non-significant. Arguably, both the brokers and the bosses are more likely negative gossip targets, at least so long as these two categories do not go together.

*Isolates* did not play any different role in gossip. As for the dyadic-level variables (the *positive* and *negative ties*), they chiefly confirmed what is expected based on the literature (see “Structural Antecedents of Gossip”). In Model 1, for example, *positive tie* (*sender–receiver*), *positive tie* (*sender–target*), and *positive tie* (*receiver–target*) have a positive association with gossip. In contrast, *negative tie* (*receiver–target*) has a negative association. These findings support that people are likely to share positive gossip with and about those they have a good relationship (McAndrew et al., 2007; Grosser et al., 2010), while they might avoid positive gossip if their receiver and target have a negative relationship with one another (Burt, 2001).

Model 2 reveals a positive association between *positive tie* (*sender–receiver*), *negative tie* (*sender–target*), *negative tie* (*receiver–target*), and negative gossip. Meanwhile, *positive tie* (*sender–target*) and *positive tie* (*receiver–target*) have a negative effect. Not surprisingly, people were more likely to share negative gossip with those they have a good relationship (Grosser et al., 2010) and about those with whom either themselves or their receiver have a troubled relationship (Wittek and Wielers, 1998; McAndrew et al., 2007; Estévez et al., 2022). In the meantime, negative gossip about friends (or friends of the receiver) was either unlikely or probably evaded (Burt, 2001).

Bewildering is the positive association between *negative tie* (*sender–receiver*) on negative gossip. It suggests that colleagues holding a negative opinion of one another were more likely to share negative gossip. One plausible explanation for this pattern is that the negative tie developed after the gossip because either specific comments about someone caused annoyance or gossip played havoc with the image of the sender (Turner et al., 2003; Gawronski and Walther, 2008; Farley et al., 2010; Caivano et al., 2020). However, the cross-sectional nature of the data does allow us to either confirm or refute this conjecture.

## Discussion and Conclusion

Every organization has its informal structure that operates more or less independently from formal relations. This structure is often fragmented into small groups characterized by relative internal cohesion and differentiation from other groups (Stadtfeld et al., 2020). These groups and the individuals who can broker between informal groups have been considered in the literature to be essential for informational flow in a firm and the transmission of gossip in particular (Gluckman, 1963; Merry, 1984; Elias and Scotson, 1994; Beersma et al., 2019). Despite all this, few studies have empirically addressed the spread of social information within/across informal groups (Tassiello et al., 2018). In the present study, we formulated hypotheses regarding the effect of informal groups and broker positions for gossip dynamics (who gossip with whom about whom). We tested our expectations using data from six firms in Hungary. Social network analysis techniques were used to identify informal groups and individuals in broker positions. Then, we used this information to predict how likely it is to observe positive or negative gossip while controlling for individual- and dyadic-level factors: gender, hierarchical position (boss vs. employee), and both positive and negative relationship ties.

Starting with the effects based on group membership, results revealed that it favors (positive and negative) gossip when the sender and receiver are members of the same informal group. This finding is consistent with a long literature suggesting that gossip is shared within groups primarily (Gluckman, 1963; Hannerz, 1967; Merry, 1984; Elias and Scotson, 1994; Kurland and Pelled, 2000; Kniffin and Wilson, 2010; Michelson et al., 2010). We also observed that individuals are more likely to send positive gossip about those in the same group. This agrees with our stated expectation (see H2a). That said, we did not observe that positive gossip about group members is shared with other group members (as captured by the null effect of *same group* (*sender–receiver–target*)). This detail raises the question of whether mechanisms other than group solidarity and signaling a commitment to in-group norms (which assume that all three gossip parties are members of the group; Ellwardt et al., 2012a) are behind the inclination to send positive gossip about group fellows. One explanation could be that, when praising group members, the sender flags their value as someone protective of their own. It is even possible that, in some cases, positive gossip attempts to enhance one’s status among out-group members rather than signal a commitment to in-group fellows (McAndrew et al., 2007).

No evidence supports the hypothesis that negative gossip concentrates on targets outside the sender’s and receiver’s group. Based on our results, whether the target is part or not of one’s informal group makes no difference at all for negative gossip. It contradicts our expectation that negative gossip avoids other group members to prevent conflicts or uncomfortable situations (Hallett et al., 2009; Giardini and Wittek, 2019b; Caivano et al., 2020). Furthermore, this finding also comes into conflict somehow with previous results in the literature. Both Kniffin and Wilson (2005) and Ellwardt et al. (2012a) noticed that negative gossip focuses on other team members. However, let us not forget that these other scholars examined row teams and formal units, where rewards demand high levels of cooperation. Unlike them, we focused on informal groups instead, where interdependencies are more affective than functional. Based on the discrepancy, one may speculate whether or not negative gossip can be an effective means of sustaining cooperation in groups that lack functional interdependencies (Feinberg et al., 2014; Giardini and Wittek, 2019a; Dores Cruz et al., 2019b). Future research may address this by comparing the impact of negative gossip on cooperation in formal vs. informal groups.

Moving to the effects based on brokerage, results support the expectations that individuals in broker positions spread more negative gossip (H4) and are more often the objects of their colleagues’ negative gossip (H6). However, no association was detected between brokerage and (positive or negative) gossip reception. Since the data here are receiver-reported (i.e., respondents reported who sent gossip to them instead whom they sent gossip to), one explanation for this null result is that many brokers could leave much gossip unreported. Perhaps for fear of disclosing information involving colleagues at distant parts of the networks or because reporting it could make them look nosy. In this regard, future studies may benefit from alternative data-collection tools. All in all, our findings concur with previous studies advocating for a more nuanced picture of brokerage, in which not everything is advantageous about this position (Krackhardt, 1999; Xiao and Tsui, 2007; Barnes et al., 2016; Tasselli and Kilduff, 2017). Here, we observed that individuals in broker positions might have to pay a high reputational price in the form of loads of negative gossip about them.

Indeed, a follow-up question could be whether this more considerable amount of negative gossip detected may be caused by brokers sending more negative gossip in the first place (or the other way around). There is evidence that individuals who are targets of negative gossip respond to it by engaging in negative gossip (Zong et al., 2021). Consequently, we cannot discard the possibility of self-reinforcing dynamics: one part starts spreading negative gossip about the other, the latter learns about it and follows suit, etc.

Since brokers have an increased relevance as senders but not receivers of negative gossip, one can speculate about the reason behind this unbalance. One possibility is that brokers have some amplifying effect: once some piece of juicy information reaches them, they send it to many others. However, it could also be that brokers are not mere transmitters but the source. Since brokers may have more leeway to exploit gossip for personal benefits (Foster and Rosnow, 2006), the increased amount of negative gossip detected in this study comes maybe from lying or making up information (Peters and Fonseca, 2020).

Our last point before addressing the limitations of this study concerns the reasons why the effect of brokers on being the target of negative gossip disappears when they are bosses. This finding was somewhat unexpected since both brokers and bosses draw lots of negative gossip upon themselves as separate categories. One plausible explanation is that when the boss holds a broker position, this tempers the inclination others have to speak negatively about them. If so, brokering can act as a buffer to the natural tendency for a boss to become the gossip target at work (Ellwardt et al., 2012c).

Like any study, ours has many limitations. Methodologically, conclusions rest on a series of techniques that are novel in the gossip literature. Many of these, however, leave space for alternative operationalization. For instance, other graph partitioning algorithms exist, which can produce different classifications (Newman and Girvan, 2004; Lancichinetti and Fortunato, 2009; Bruggeman et al., 2012). Likewise, alternative metrics could be used to capture a subject’s brokerage potential, including betweenness in a weighted rather than binary network (Opsahl et al., 2010). We always tried to use widely accepted measures as a rule of thumb, but agreement on which measure is best could change. On a similar note, different types of ties can be distinguished beyond simply positive vs. negative (for instance, expressive vs. instrumental; see Umphress et al., 2003) and used as the basis for group detection algorithms. Finally, note that this study does not control for personality traits or psychological factors, although we partly attributed why brokers send negative gossip to a self-monitoring personality (Sasovova et al., 2010; Landis, 2016).

Taken together, our results demonstrate that structural aspects beyond the relationship ties in the gossip triad (Wittek and Wielers, 1998; Giardini and Wittek, 2019b) matter for workplace gossip dynamics. In studying some of these aspects (*viz.* informal groups and network brokerage), we gained further insights into the specific contexts where negative gossip is more likely a viable solution for the hassle of sustaining cooperation. Importantly, our findings suggest that brokers can use their structural position to control the social information in the organization. Yet, perhaps because of this, they are also subject to the negative evaluations of their colleagues.

## Data Availability Statement

The datasets supporting the present findings of this document including as a replication package and all code used in the article is readily available via https://github.com/joseluisesna/Gossip_in_Hungarian_firms.

## Ethics Statement

The studies involving human participants were reviewed and approved by Research Ethics Committee of the Centre for Social Sciences, Budapest. The participants provided their written informed consent to participate in this study.

## Author Contributions

KT designed and supervised data collection and commented and worked on sections resulting in the current manuscript. JE developed the theoretical framework, performed statistical analyses, and wrote the first draft of the manuscript. All authors contributed to the article and approved the submitted version.

## Funding

This research has been supported by the European Research Council (ERC) under the European Union’s Horizon 2020 research and innovation program (grant agreement no. 648693, PI: KT). KT thanks the support of the National Research, Development and Innovation Office—NKFIH (OTKA) grant K 132250, PI: Szabolcs Számadó, and JE thanks the support of “la Caixa” Foundation, Spain [ID 100010434 (fellowship code LCF/BQ/EU17/115900700)].

## Conflict of Interest

The authors declare that the research was conducted in the absence of any commercial or financial relationships that could be construed as a potential conflict of interest.

## Publisher’s Note

All claims expressed in this article are solely those of the authors and do not necessarily represent those of their affiliated organizations, or those of the publisher, the editors and the reviewers. Any product that may be evaluated in this article, or claim that may be made by its manufacturer, is not guaranteed or endorsed by the publisher.

## Footnotes

^1^Further information about the sample is available in Supplementary Table S1.

^2^Supplementary Table S2 contains all the variables used in the current study.

^3^Two items were excluded: “Are the executive pet” and “I want to make better than them.”

^4^Though some positive items emphasize affective aspects whereas others capture more instrumental ones (Lincoln and Miller, 1979; Umphress et al., 2003), these two aspects are closely intertwined in our data. Therefore, we did not distinguish between sub-types of positive ties in this article (e.g., a three- or four-dimension solution).

^5^Still, four respondents in Unit C had their out-going positive ties corrected. This is because of their seemingly indiscriminate nomination of others (22–28 ties sent or (almost) all colleagues). For these four respondents, we only kept ties when there are mutual (i.e., if *i* nominates *j* and *j* chooses *i*). A total of 78 ties were deleted.

^6^A further description of the positive and negative networks is available in Supplementary Tables S3, S4.

^7^Supplementary Tables S3, S4 shows that, while in units B, C, D and E, individuals send on overage 3.2–4.9 positive and 0.4–0.9 negative ties, in units A and F these same numbers are 2.0–2.2 and 1.0–1.9, respectively.

^8^Supplementary Figure S1 displays a classification of all the gossip triads in our data by group membership and unit.
