# Supplementary material for: Brokering or Sitting Between Two Chairs? A Group Perspective on Workplace Gossip
Source: Front Psychol. 2022 Jul 8;13:815383. doi: 10.3389/fpsyg.2022.815383 (PMC9309222; doi:10.3389/fpsyg.2022.815383)
Supplement: Supplementary file 1 [file Data_Sheet_1.docx]

***Supplementary Material***

**Table S1. Summary statistics**

|  | **Unit A** | **Unit B** | **Unit C** | **Unit D** | **Unit E** | **Unit F** |
| --- | --- | --- | --- | --- | --- | --- |
| Sector | Public Sector | Web Dev. | Software Dev. | Access Control | Software Dev. | Finance |
| Unit members | 24 | 19 | 29 | 18 | 16 | 22 |
| Women | 18 | 5 | 1 | 6 | 10 | 1 |
| Bosses | 6 | 6 | 4 | 6 | 8 | 4 |
| Female bosses | 4 | 3 | 0 | 1 | 2 | 0 |
| Average age | 37.7 | 31.7 | 32.8 | 39.4 | 39.6 | 37.9 |
| Age (range) | 25-55 | 24-42 | 22-42 | 25-64 | 24-56 | 28-49 |
| Average tenure | 2.3 | 3.8 | 2.2 | 4.1 | 2.2 | – |
| Tenure (range) | 1.1-5.6 | 1.3-7.4 | 0.6-4.6 | 0.7-16.9 | 0.3-4.5 | – |
| Note: Unit F did not provide information regarding personnel’s tenure or enrolment date. | | | | | | |

**Table S2. Overview of the variables used in the study**

| **Variable** | **Measurement** | **Name in the dataset** |
| --- | --- | --- |
| ID | Identifier | responder |
| Gender | Woman = 1 | woman |
| Firm | Firm identifier | org |
| Managerial position | Manager = 1 | hr_leader |
| Age | Year of birth | birth_year |
| Tenure | Enrolment date | hr_work_start |
| ***Sociometric variables*** | | |
| Are a friend | Nomination | friend |
| Are trustworthy | Nomination | trustworthy |
| Regular personal conversations | Nomination | personal_conversation_ |
| Are neutral to me | Nomination | is_neutral |
| Cooperate with job duties | Nomination | cooperate_job_duties |
| Regular work conversations | Nomination | work_conversation_ |
| Are not a friend | Nomination | not_friend |
| Share negative information about me | Nomination | shares_negative_info_abou_me |
| Are the executive pet | Nomination | executive_pet |
| Would not cooperate | Nomination | would_not_cooperate |
| Do not belong to the team | Nomination | belong_to_team |
| I want to be better than them | Nomination | want_to_be_better_than_her |
| I appreciate them^1^ | Nomination | appreciation |
| I turn for help | Nomination | turn_for_her_help |
| Cooperate well | Nomination | cooperate_well |
| I listen to them | Nomination | listen_to_her |
| Do their job well | Nomination | does_job_well |
| Are not suitable for the job | Nomination | not_suitable_for_job |
| Are popular | Nomination | popular |
| Deserve wage increase | Nomination | wage_increasing |
| Deserve wage cut | Nomination | wage_reduction |
| Other colleagues appreciate them | Nomination | colleagues_appreciate |
| Other colleagues ask them for help | Nomination | colleagues_ask_for_her_help |
| Other colleagues listen to them | Nomination | colleagues_listen_to_her |
| Other colleagues despise them | Nomination | colleagues_despise |
| ***Gossip***^2^ |  |  |
| Sender (who gave you personal information about another colleague) | Nomination | sender |
| Target (about whom) | Nomination | target |
| Tone (negative, neutral, positive) | [–1,0,1] | info |
| Note:  ^1^ Inspired in Laird *et al.* (2012).  ^2^ Inspired in Ellwardt *et al.* (2012). | | |

**Table S3. Summary statistics of the network of positive ties**

|  | **Unit A** | **Unit B** | **Unit C** | **Unit D** | **Unit E** | **Unit F** |
| --- | --- | --- | --- | --- | --- | --- |
| Nodes | 24 | 19 | 29 | 18 | 16 | 22 |
| Missing tie | 0.0% | 0.0% | 13.3% | 0.0% | 0.0% | 4.5% |
| Density | 8.5% | 22.8% | 17.5% | 21.2% | 21.2% | 10.6% |
| Reciprocity | 25.5% | 35.9% | 45.1% | 49.2% | 54.9% | 24.5% |
| Transitive closure | 36.8% | 50.0% | 47.0% | 45.1% | 57.5% | 35.6% |
| Isolates | 3 | 0 | 1 | 1 | 1 | 2 |
| Ave. degree | 2.0 | 4.1 | 4.9 | 3.6 | 3.2 | 2.2 |
| SD out-degree | 1.8 | 3.1 | 4.1 | 2.1 | 2.9 | 2.3 |
| SD in-degree | 2.4 | 2.7 | 3.7 | 3.5 | 1.9 | 2.1 |

**Table S4. Summary statistics of the network of negative ties**

|  | **Unit A** | **Unit B** | **Unit C** | **Unit D** | **Unit E** | **Unit F** |
| --- | --- | --- | --- | --- | --- | --- |
| Nodes | 24 | 19 | 29 | 18 | 16 | 22 |
| Missing tie | 0.0% | 0.0% | 10.3% | 0.0% | 0.0% | 4.5% |
| Density | 4.3% | 5.0% | 1.4% | 4.9% | 5.0% | 8.9% |
| Reciprocity | 16.7% | 0.0% | 0.0% | 0.0% | 0.0% | 14.6% |
| Transitive closure | 21.1% | 7.5% | 0.0% | 7.0% | 17.1% | 23.5% |
| Isolates | 9 | 5 | 18 | 5 | 5 | 2 |
| Ave. degree | 1.0 | 0.9 | 0.4 | 0.8 | 0.8 | 1.9 |
| SD out-degree | 1.2 | 0.9 | 0.8 | 0.9 | 0.9 | 2.4 |
| SD in-degree | 1.4 | 1.6 | 1.1 | 2.1 | 2.0 | 2.3 |

**Table S5: Multilevel logistic unstandardised estimates of the association between same-group membership/brokerage and positive gossip**

|  | **Null Model** | | | **Model 1** | | | **Model 2** | | | **Model 3** | | |
| --- | --- | --- | --- | --- | --- | --- | --- | --- | --- | --- | --- | --- |
|  | **Est.** | **SE** |  | **Est.** | **SE** |  | **Est.** | **SE** |  | **Est.** | **SE** |  |
| **Fixed factors** |  |  |  |  |  |  |  |  |  |  |  |  |
| (Intercept) | -7.98 | 0.40 | *** | -9.56 | 0.50 | *** | -9.81 | 0.50 | *** | -9.95 | 0.51 | *** |
| ***Individual-level control variables*** |  |  |  |  |  |  |  |  |  |  |  |  |
| Woman (sender) |  |  |  | 0.39 | 0.36 |  | 0.32 | 0.36 |  | 0.30 | 0.35 |  |
| Woman (receiver) |  |  |  | 0.22 | 0.60 |  | 0.18 | 0.60 |  | -0.01 | 0.60 |  |
| Woman (target) |  |  |  | 0.01 | 0.21 |  | -0.07 | 0.21 |  | 0.01 | 0.21 |  |
| Boss (sender) |  |  |  | 1.29 | 0.32 | *** | 1.22 | 0.31 | *** | 1.03 | 0.33 | ** |
| Boss (receiver) |  |  |  | 1.43 | 0.59 | * | 1.48 | 0.58 | * | 1.31 | 0.62 | * |
| Boss (target) |  |  |  | -0.06 | 0.19 |  | -0.04 | 0.19 |  | -0.03 | 0.21 |  |
| Isolate (sender) |  |  |  |  |  |  |  |  |  | 0.06 | 0.66 |  |
| Isolate (receiver) |  |  |  |  |  |  |  |  |  | 1.52 | 1.10 |  |
| Isolate (target) |  |  |  |  |  |  |  |  |  | -0.96 | 0.49 |  |
| ***Dyadic-level control variables*** |  |  |  |  |  |  |  |  |  |  |  |  |
| Positive tie (sender-receiver) |  |  |  | 1.66 | 0.15 | *** | 1.30 | 0.16 | *** | 1.24 | 0.16 | *** |
| Positive tie (sender-target) |  |  |  | 1.04 | 0.13 | *** | 0.75 | 0.14 | *** | 0.72 | 0.14 | *** |
| Positive tie (receiver-target) |  |  |  | 0.84 | 0.13 | *** | 0.67 | 0.14 | *** | 0.68 | 0.14 | *** |
| Negative tie (sender-receiver) |  |  |  | -0.15 | 0.33 |  | -0.09 | 0.33 |  | -0.13 | 0.33 |  |
| Negative tie (sender-target) |  |  |  | -0.86 | 0.40 | * | -0.81 | 0.40 | * | -0.77 | 0.40 |  |
| Negative tie (receiver-target) |  |  |  | -0.80 | 0.34 | * | -0.78 | 0.34 | * | -0.69 | 0.34 | * |
| ***Variables based on group membership*** |  |  |  |  |  |  |  |  |  |  |  |  |
| Same group (sender-receiver) [**H1a**] |  |  |  |  |  |  | 0.66 | 0.17 | *** | 0.69 | 0.17 | *** |
| Same group (sender-target) [**H2a**] |  |  |  |  |  |  | 0.64 | 0.16 | *** | 0.62 | 0.16 | *** |
| Same group (receiver-target) [**H3a**] |  |  |  |  |  |  | 0.28 | 0.19 |  | 0.26 | 0.19 |  |
| Same group (sender-receiver-target) |  |  |  |  |  |  | -0.06 | 0.32 |  | -0.05 | 0.32 |  |
| ***Variables based on broker status*** |  |  |  |  |  |  |  |  |  |  |  |  |
| Broker (sender) |  |  |  |  |  |  |  |  |  | 0.54 | 0.34 |  |
| Broker (receiver) [**H5a**] |  |  |  |  |  |  |  |  |  | 0.89 | 0.65 |  |
| Broker (target) |  |  |  |  |  |  |  |  |  | -0.11 | 0.21 |  |
| **Random factors** |  |  |  |  |  |  |  |  |  |  |  |  |
| Unit (SD) | <0.01 |  |  | 0.01 |  |  | 0.01 |  |  | 0.02 |  |  |
| Sender (SD) | 1.44 |  |  | 1.36 |  |  | 1.32 |  |  | 1.27 |  |  |
| Receiver (SD) | 2.69 |  |  | 2.49 |  |  | 2.48 |  |  | 2.41 |  |  |
| Target (SD) | 0.75 |  |  | 0.65 |  |  | 0.67 |  |  | 0.66 |  |  |
| **Model fit** |  |  |  |  |  |  |  |  |  |  |  |  |
| Log likelihood | -2,129 |  |  | -1,984 |  |  | -1,955 |  |  | -1,950 |  |  |
| Deviance | 4,258 |  |  | 3,967 |  |  | 3,910 |  |  | 3,900 |  |  |
| ANOVA (df) |  |  |  | 290.28 | (12) | *** | 57.40 | (4) | *** | 10.21 | (6) |  |
| Note: * *p* < .05, ** *p* < .01, *** *p* < .001; two-tailed test.  The ANOVA tests whether the model fit improved with the addition of extra predictors. | | | | | | | | | | | | |

**Table S6: Multilevel logistic unstandardised estimates of the association between same-group membership/brokerage and negative gossip**

|  | **Null Model** | | | **Model 1** | | | **Model 2** | | | **Model 3** | | |
| --- | --- | --- | --- | --- | --- | --- | --- | --- | --- | --- | --- | --- |
|  | **Est.** | **SE** |  | **Est.** | **SE** |  | **Est.** | **SE** |  | **Est.** | **SE** |  |
| **Fixed factors** |  |  |  |  |  |  |  |  |  |  |  |  |
| (Intercept) | -9.15 | 0.59 | *** | -10.18 | 0.58 | *** | -10.30 | 0.58 | *** | -10.56 | 0.61 | *** |
| ***Individual-level control variables*** |  |  |  |  |  |  |  |  |  |  |  |  |
| Woman (sender) |  |  |  | 0.15 | 0.38 |  | 0.06 | 0.38 |  | 0.07 | 0.39 |  |
| Woman (receiver) |  |  |  | 0.34 | 0.62 |  | 0.42 | 0.62 |  | 0.55 | 0.66 |  |
| Woman (target) |  |  |  | -0.18 | 0.28 |  | -0.21 | 0.28 |  | -0.36 | 0.29 |  |
| Boss (sender) |  |  |  | 1.28 | 0.33 | *** | 1.29 | 0.33 | *** | 1.01 | 0.35 | ** |
| Boss (receiver) |  |  |  | 2.20 | 0.63 | *** | 2.17 | 0.62 | *** | 2.11 | 0.70 | ** |
| Boss (target) |  |  |  | 0.76 | 0.25 | ** | 0.76 | 0.24 | ** | 0.59 | 0.26 | * |
| Isolate (sender) |  |  |  |  |  |  |  |  |  | 0.21 | 0.65 |  |
| Isolate (receiver) |  |  |  |  |  |  |  |  |  | -1.30 | 1.33 |  |
| Isolate (target) |  |  |  |  |  |  |  |  |  | 0.69 | 0.41 |  |
| ***Dyadic-level control variables*** |  |  |  |  |  |  |  |  |  |  |  |  |
| Positive tie (sender-receiver) |  |  |  | 1.25 | 0.17 | *** | 0.91 | 0.18 | *** | 0.88 | 0.19 | *** |
| Positive tie (sender-target) |  |  |  | -0.55 | 0.20 | ** | -0.47 | 0.22 | * | -0.54 | 0.22 | * |
| Positive tie (receiver-target) |  |  |  | -0.58 | 0.20 | ** | -0.71 | 0.21 | *** | -0.73 | 0.21 | *** |
| Negative tie (sender-receiver) |  |  |  | 1.06 | 0.23 | *** | 1.18 | 0.23 | *** | 1.16 | 0.23 | *** |
| Negative tie (sender-target) |  |  |  | 1.37 | 0.17 | *** | 1.33 | 0.18 | *** | 1.34 | 0.18 | *** |
| Negative tie (receiver-target) |  |  |  | 1.22 | 0.17 | *** | 1.25 | 0.17 | *** | 1.26 | 0.17 | *** |
| ***Variables based on group membership*** |  |  |  |  |  |  |  |  |  |  |  |  |
| Same group (sender-receiver) [**H1b**] |  |  |  |  |  |  | 0.57 | 0.17 | *** | 0.59 | 0.17 | *** |
| Same group (sender-target) ) [**H2b**] |  |  |  |  |  |  | -0.32 | 0.22 |  | -0.26 | 0.22 |  |
| Same group (receiver-target) ) [**H3b**] |  |  |  |  |  |  | 0.31 | 0.19 |  | 0.34 | 0.20 |  |
| Same group (sender-receiver-target) |  |  |  |  |  |  | 0.35 | 0.37 |  | 0.31 | 0.37 |  |
| ***Variables based on broker status*** |  |  |  |  |  |  |  |  |  |  |  |  |
| Broker (sender) [**H4**] |  |  |  |  |  |  |  |  |  | 0.78 | 0.36 | * |
| Broker (receiver) [**H5b**] |  |  |  |  |  |  |  |  |  | 0.19 | 0.72 |  |
| Broker (target) [**H6**] |  |  |  |  |  |  |  |  |  | 0.66 | 0.27 | * |
| **Random factors** |  |  |  |  |  |  |  |  |  |  |  |  |
| Unit (SD) | <0.01 |  |  | 0.03 |  |  | 0.01 |  |  | 0.01 |  |  |
| Sender (SD) | 1.57 |  |  | 1.34 |  |  | 1.35 |  |  | 1.32 |  |  |
| Receiver (SD) | 3.12 |  |  | 2.58 |  |  | 2.57 |  |  | 2.65 |  |  |
| Target (SD) | 1.26 |  |  | 0.88 |  |  | 0.88 |  |  | 0.85 |  |  |
| **Model fit** |  |  |  |  |  |  |  |  |  |  |  |  |
| Log likelihood | -1,798 |  |  | -1,680 |  |  | -1,667 |  |  | -1,660 |  |  |
| Deviance | 3,596 |  |  | 3,359 |  |  | 3,334 |  |  | 3,320 |  |  |
| ANOVA (df) |  |  |  | 236.8 | (12) | *** | 25.5 | (4) | *** | 13.4 | (6) | * |
| Note: * *p* < .05, ** *p* < .01, *** *p* < .001; two-tailed test.  The ANOVA tests whether the model fit improved with the addition of extra predictors. | | | | | | | | | | | | |

**Figure S1: Distribution of the gossip triads by group membership and unit**


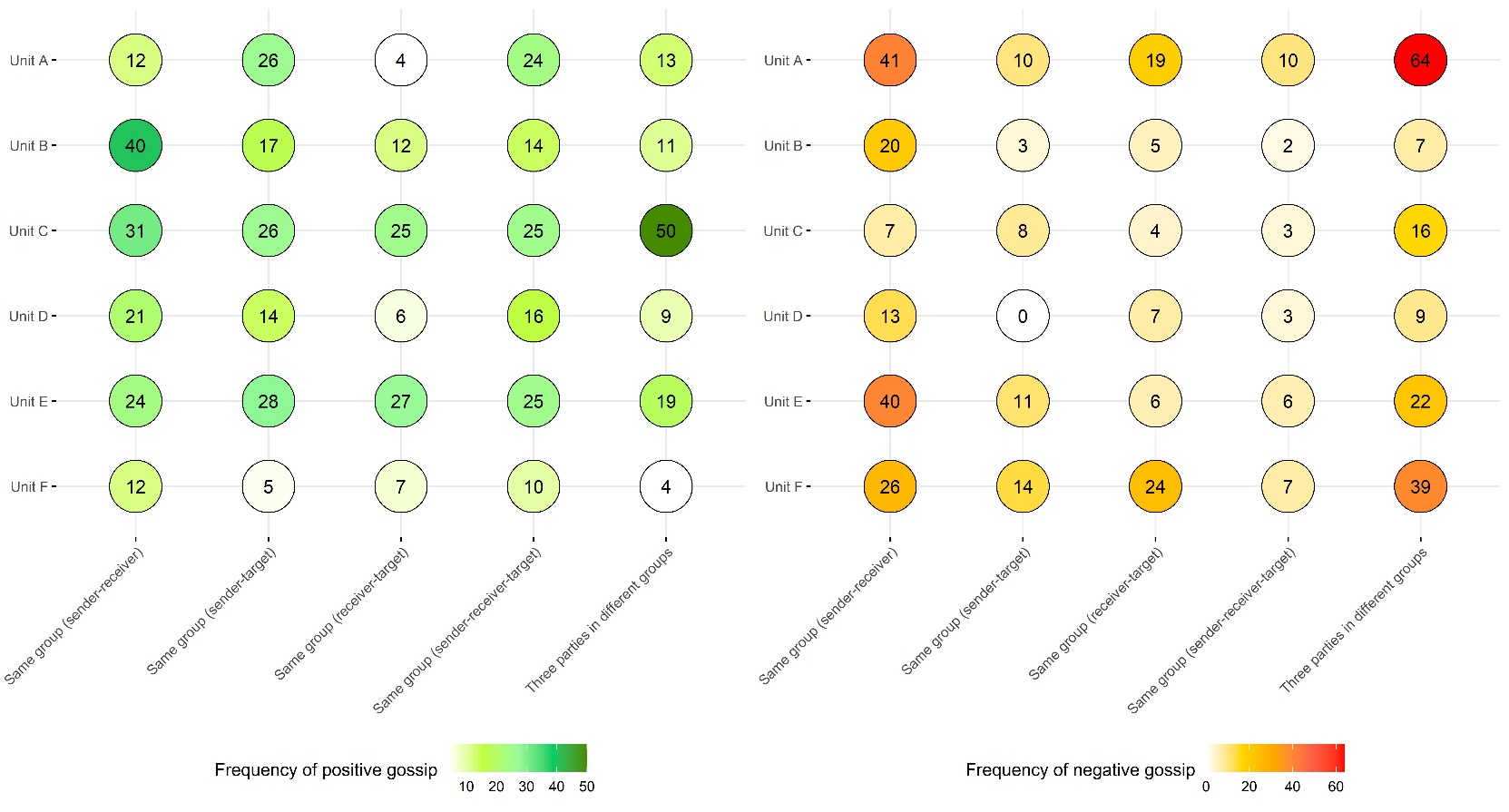


**REFERENCES**

Ellwardt, L., Labianca, G. (Joe), and Wittek, R. (2012). Who are the objects of positive and negative gossip at work?: A social network perspective on workplace gossip. *Soc. Netw.* 34, 193–205. doi: 10.1016/j.socnet.2011.11.003.

Laird, M. D., Zboja, J. J., and Ferris, G. R. (2012). Partial mediation of the political skill-reputation relationship. *Career Dev. Int.* 17, 557–582. doi: 10.1108/13620431211280132.
